# Low-Intensity Pulsed Ultrasound in Peripheral and Central Nerve Repair: Mechanisms and Emerging Therapeutic Strategies

**DOI:** 10.3390/jfb17030113

**Published:** 2026-02-26

**Authors:** Cheng Ma, Saijie Song, Jianwu Dai, He Shen

**Affiliations:** 1 State Key Laboratory of Advanced Medical Materials and Devices, Tianjin Key Laboratory of Biomedical Materials, Tianjin Key Laboratory of Neuromodulation and Neurorepair, Institute of Biomedical Engineering, Tianjin Institutes of Health Science, Chinese Academy of Medical Science & Peking Union Medical College, Tianjin 300192, China; macheng@bme.pumc.edu.cn; 2 State Key Laboratory of Microbial Technology, School of Food Science and Pharmaceutical Engineering, Nanjing Normal University, Nanjing 210023, China; ssj2016@mail.ustc.edu.cn

**Keywords:** low-intensity pulsed ultrasound, neural regeneration and repair, therapeutic ultrasound, mechanotransduction, smart biomaterial-assisted neuromodulation

## Abstract

Low-intensity pulsed ultrasound (LIPUS) has emerged as a versatile, non-invasive physical modality with growing potential in regenerative medicine and neural repair. Advances in ultrasound physics and biomedical engineering have enabled precise spatiotemporal control of acoustic stimulation, positioning therapeutic ultrasound as an alternative to conventional pharmacological and surgical interventions that often suffer from limited targeting and substantial side effects. Unlike high-intensity focused ultrasound, which relies primarily on thermal ablation, LIPUS operates within a low-energy, non-thermal regime and modulates cellular behavior through mechanical cues, mechano-transduction, and downstream biological responses. Accumulating evidence demonstrates that LIPUS regulates calcium dynamics, cytoskeletal remodeling, neurotrophic factor expression, inflammation, myelination, and local vascular remodeling, thereby promoting functional recovery in both peripheral and central nerve injury models. Moreover, the integration of LIPUS with biomaterials, including piezoelectric scaffolds and acoustically responsive drug delivery systems, has expanded its functionality from direct stimulation to on-demand electrical signaling and controlled therapeutic release. Despite these advances, challenges remain regarding parameter standardization, mechanistic consistency, and clinical translation. In this review, we summarize the systems, parameters, and biological mechanisms underlying LIPUS, discuss its applications in peripheral and central nerve injury repair, and highlight emerging strategies and translational barriers toward intelligent, multimodal, and personalized ultrasound-based therapies.

## 1. Introduction

The late 20th and early 21st centuries have witnessed significant advancements in disciplines such as biomedical engineering, physics, computer science, and artificial intelligence, which have prompted the emergence, development, and widespread application of numerous novel diagnostic and therapeutic techniques. Progress in acoustic technology, particularly in ultrasound physics, has deepened interdisciplinary research between biomedicine and physics, driving innovation in medical methods such as high-resolution diagnostic imaging and non-invasive targeted therapy.

In clinical practice, ultrasound is typically applied in two main domains: diagnostic ultrasound and therapeutic ultrasound [1]. Diagnostic ultrasound is a non-invasive in vivo imaging technique commonly used clinically for disease diagnosis. It enables the non-invasive acquisition of images of internal organs or other structures, information on blood flow velocity, tissue stiffness, and other physical characteristics, thereby visualizing diseases in tissues or organs. Therapeutic ultrasound aims to interact with cells, tissues, and organs within the body, or with materials implanted in the body, to achieve functions such as destroying abnormal tissue, tissue regeneration, or promoting drug release, ultimately for disease treatment [1,2,3]. This review primarily focuses on discussing therapeutic ultrasound and the combined role of ultrasound and acoustic-responsive materials in disease control or treatment.

**Figure 1:**
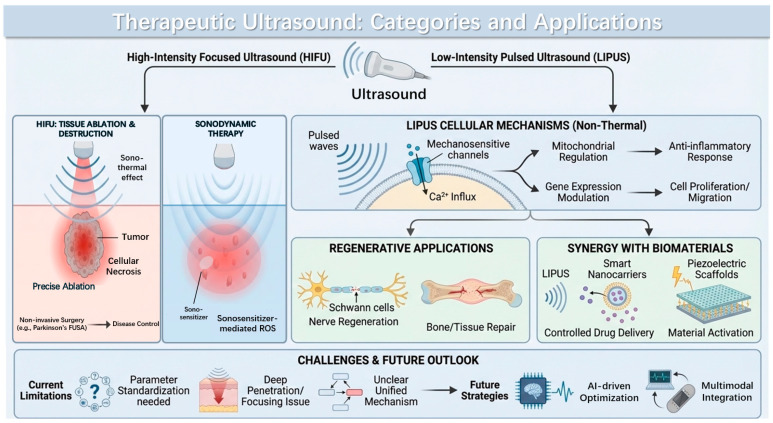
Categories and biomedical applications of therapeutic ultrasound. Schematic overview illustrating the distinct working principles of high-intensity focused ultrasound (HIFU) and low-intensity pulsed ultrasound (LIPUS), together with their representative applications, current limitations, and future perspectives.

It is undeniable that traditional treatment modalities, including pharmacotherapy and surgery, remain mainstream within the current healthcare system. However, these conventional approaches often have inherent drawbacks. For instance, in pharmacotherapy, systemic drug distribution throughout the body makes localized, precise targeting difficult, resulting in limited efficacy and significant side effects; long-term use may also lead to drug resistance or affect multiple organ functions. While surgery can provide direct and controllable intervention to some extent, its inherently invasive nature brings issues such as tissue damage, infection risks, postoperative pain, and lengthy recovery times, and not all patients are suitable candidates for surgery. Therefore, there is a pressing clinical need for a novel physical intervention that is non-invasive, precise, and capable of personalized modulation to reduce side effects and improve targeting. In light of the aforementioned issues, therapeutic ultrasound has gradually garnered attention. Most therapeutic ultrasound modalities are non-invasive, requiring no incisions or lacerations on the skin during treatment, so no scars are left on the skin during its recovery period. Moreover, due to its deep tissue penetration, real-time adjustability, and good safety profile, it is considered an ideal strategy to achieve this goal. Ultrasound can be classified in many ways, with common categories primarily divided into two types: high-intensity focused ultrasound (HIFU) and low-intensity pulsed ultrasound (LIPUS) (Figure 1).

HIFU, which is FDA-approved for the treatment of uterine fibroids, can achieve precise destruction of internal tumors or other abnormal tissues without creating wounds or damaging surrounding tissues, enabling it to serve as a powerful adjunct to existing treatment modalities [4,5]. Studies have also shown that HIFU can be used for hemostasis or ablating blood clots [6,7,8]. This non-invasive treatment approach increases patient compliance to some extent, reduces patient suffering during treatment while ensuring therapeutic efficacy, and aligns better with contemporary medical philosophy. In fact, as a treatment modality, having sufficiently excellent efficacy is paramount. Another characteristic of ultrasound therapy is its ability to provide precise targeting and adjustable treatment [9,10,11], meaning that by adjusting ultrasound parameters such as frequency, intensity, or focusing methods, energy can be precisely concentrated in specific treatment areas. Parameters can also be tuned according to different needs to achieve ablation of abnormal tissue, activation of specific biomaterials, or precise drug release. To some extent, in terms of precision and targeting, ultrasound therapy can rival the accuracy of surgery. Therefore, owing to its non-invasive nature, it holds the potential to replace traditional surgical methods in the future, enabling precise treatment of the affected area. Furthermore, the controllability of ultrasound is also reflected in its treatment depth and range, allowing ultrasound therapy to cover deeper tissues [12,13], demonstrating significant application potential, particularly in treating deep-seated organs such as the spinal cord and brain [14,15,16]. A highly compelling example is focused ultrasound ablation (FUSA), a technique approved by the U.S. Food and Drug Administration (FDA) for treating Parkinson’s disease, which can target and ablate specific brain structures and is a non-invasive medical treatment technology. A clinical study on focused ultrasound in Parkinson’s disease concluded that ablation of the internal segment of the globus pallidus can alleviate motor symptoms in Parkinson’s patients [17]. Thus, it can be demonstrated that ultrasound therapy is capable of achieving personalized treatment that is non-invasive, precisely targeted, and controllable in terms of depth and treatment range.

As a form of energy capable of penetrating deep tissues, ultrasound technology is widely applied in various biomedical scenarios. From an application aspect, LIPUS and HIFU exhibit fundamental differences in both operational intensity ranges and primary biological effects. (Table 1) HIFU employs focused ultrasound, with focal intensities typically around 100 W/cm^2^ and locally reaching up to several kilowatts per square centimeter, thereby enabling rapid temperature elevation and subsequent localized tissue necrosis. Accordingly, the primary mechanism of HIFU is thermal ablation. In contrast, LIPUS operates at substantially lower intensities than HIFU, typically 30–100 mW/cm^2^ and generally below 3 W/cm^2^, delivered in pulsed mode to avoid significant thermal effects. Consequently, the biological effects of LIPUS are achieved predominantly through mechanical stimulation and mechano-transduction rather than thermal mechanisms. Thus, focused ultrasound has made significant progress in areas such as tumor ablation, whereas LIPUS leverages its superior biosafety and is more suitable for promoting tissue regeneration, modulating cell behavior, regulating drug release from biomaterials or piezoelectric effects, and improving the internal microenvironment [18,19,20,21,22,23,24]. In recent years, an increasing number of studies have regarded LIPUS as an ideal tool in regenerative medicine for various scenarios such as nerve injury, fracture healing, and inflammation regulation.

In fact, numerous current studies based on therapeutic ultrasound are continually emerging, all aiming to investigate the mechanisms by which different types and parameters of ultrasound stimulation contribute to disease treatment, including but not limited to acoustic mechanical stimulation, acoustic thermal effects, acoustoelectric effects, and the impact of acoustic stimulation on cells or tissues. In the fields of regeneration and cell behavior modulation, although LIPUS demonstrates exciting potential, current research still presents several contradictions: first, a unified theory on the biological mechanisms of LIPUS has not been established, with different studies emphasizing different primary pathways, such as Piezo channels [30], Ca^2+^ signaling [31], mitochondrial regulation [32], or inflammation suppression [33,34]. Second, significant variations exist in acoustic parameters (e.g., frequency, intensity, duty cycle, and irradiation time) across studies, leading to poor comparability of results. Third, the responses between different disease models are not entirely consistent, necessitating further validation of their translational potential. Additionally, LIPUS still faces bottlenecks in deep tissue attenuation, energy control, precise focusing, and clinical parameter standardization. With the ongoing integration of LIPUS with new technologies such as biomaterials, AI-based intelligent control, and multimodal stimulation in recent years, it is necessary to critically review current evidence, summarize key issues, and provide direction and strategies for subsequent translational research.

Based on this, this review aims to provide a comprehensive overview of LIPUS -an ultrasound treatment modality with low tissue damage risk and lower incidence of adverse reactions—its equipment, parameters, biological mechanisms, and the application progress of LIPUS in tissue engineering, especially in the field of PNI/CNI repair. Unlike previous narrative summaries, this review aims to comprehensively reorganize existing LIPUS tissue repair studies into an experiment-oriented framework that integrates parameter selection, biological mechanisms prediction, and translational consideration, thereby providing practical guidance for further experiment design. This review will delve into its mechanisms of action, analyze potential methodological limitations and data contradictions in current development, and discuss its future prospects and translational barriers in combination with new technologies.

## 2. LIPUS System, Parameters, and Mechanisms of Action

### 2.1. LIPUS System

LIPUS is an acoustic treatment modality that utilizes low-intensity and intermittent acoustic pulses for stimulation, exhibiting positive effects on the regeneration and anti-inflammation of biological tissues such as bone, cartilage, tendon, and nerve [33,34]. Its core advantages lie in its non-invasiveness, precise treatment localization, ability to penetrate deep tissues, tunable parameters, and minimal thermal damage. In recent years, due to its safety and repeatable application characteristics, LIPUS has facilitated more research and applications in the fields of regenerative medicine, nerve repair, and inflammation regulation. In the current medical field, LIPUS is also commonly employed as a potent method for physical therapy.

**Figure 2:**
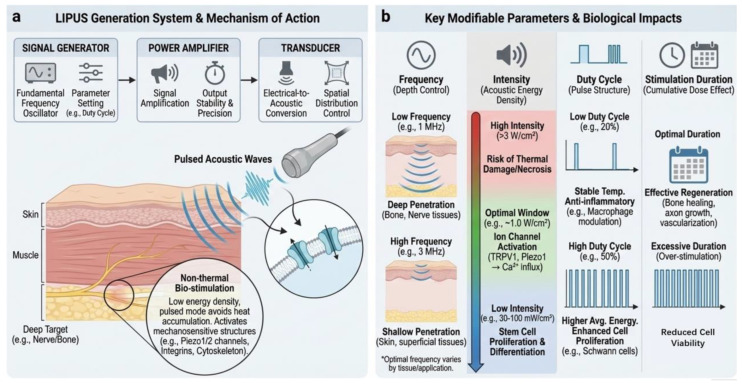
The LIPUS therapeutic platform: system components and parameter-dependent biological effects. (**a**) Schematic illustration of a representative LIPUS generation system and the underlying mechanisms of non-thermal bio-stimulation. (**b**) Key modifiable LIPUS parameters and their associated biological responses.

Whether at the clinical or research level, applying LIPUS stimulation typically requires a complete LIPUS system. A typical LIPUS system comprises three core components: a signal generator, a power amplifier & pulsing controller, and a transducer [35]. The signal generator is used to produce the fundamental acoustic waveform. Within it, the fundamental frequency oscillator delivers therapeutic ultrasound waves and determines the operating frequency of LIPUS [36]. Additionally, the signal generator also incorporates functions for parameter setting (e.g., duty cycle, etc.). This component determines the functionality achievable by LIPUS; for instance, relatively low-frequency ultrasound possesses stronger tissue penetration capability, enabling therapeutic effects on deep tissues. The power amplifier is responsible for amplifying the signal into therapeutic energy levels and ensuring stable and precise output. This ensures that the LIPUS signal can safely penetrate deep tissues and maintain stable energy density. The transducer functions to convert electrical signals into ultrasound waves. It determines the spatial distribution of energy within tissues and the penetration depth. These three parts collectively determine the physical characteristics, controllability, tissue targeting, and ultimately the nerve repair efficacy of LIPUS (Figure 2a).

Through the synergistic action of the aforementioned three main modules, the LIPUS system can deliver physical signals with relatively low energy density, offering relatively high safety. Therefore, its intensity is typically below the threshold for causing thermal damage, and its pulsed mode prevents continuous acoustic energy accumulation, making LIPUS treatment less prone to thermal effects and thus not leading to tissue necrosis. Compared to FUS, LIPUS is more suitable for tissue repair scenarios requiring long-term, repeated stimulation [37,38]. The gentler stimulation mode maximizes the effect of mechanical forces on cells, facilitating the activation of cellular mechanosensitive structures such as Piezo1/2 channels, integrins, and the cytoskeleton. (Figure 2a) Furthermore, the adjustability of LIPUS parameters, including power, duty cycle, and total stimulation time, renders its physical signal source customizable, facilitating optimization for different pathological states and tissue depths. This also provides an important prerequisite for its combination with acoustic-responsive materials, such as acoustically responsive hydrogels, nanoparticles, or piezoelectric materials, granting it greater flexibility in biomaterial-assisted tissue regeneration and inflammation regulation.

### 2.2. Key Parameters and Biological Impacts of LIPUS

As mentioned earlier, when LIPUS is applied to stimulate different pathological states and tissue depths, its therapeutic efficacy is highly dependent on acoustic parameters, encompassing but not limited to changes in intracellular reactive oxygen species levels, regulation of inflammation, release of cytokines, and control of material piezoelectricity, among others. Therefore, this section will primarily discuss a series of parameters related to LIPUS and their biological impacts.

#### 2.2.1. Frequency

Frequency determines the propagation behavior of ultrasound in tissue, influencing factors such as tissue penetration depth and spatial resolution. Generally, higher frequencies can improve resolution but relatively reduce penetration capability, whereas lower frequencies enhance penetration at the cost of lower resolution [39]. Feng et al. demonstrated in their study that the penetration depth of 1 MHz ultrasound in tissue is approximately 4 cm (muscle) to 0.058 cm (bone), while that of 3 MHz ultrasound is about 1.1 cm (muscle) to 0.006 cm (bone) [40]. This also indicates that for stimulating deep tissues, such as in central nerve injury (CNI) and peripheral nerve injury (PNI), lower-frequency LIPUS is relatively more suitable. Conversely, relatively higher-frequency stimulation is more applicable for superficial stimulation, such as in skin repair or in vitro cell experiments (Figure 2b).

Studies have also shown that ultrasound of different frequencies can have varying effects on tissue repair functionality. In an experiment on rabbit mandibular bone defects, 3 MHz LIPUS was found to possess higher biological efficacy in promoting bone healing [41]. Similar results were validated in a rat alveolar bone healing model [42]. It should be noted that this conclusion has not been verified in all studies; some investigations have found no difference in the promotive effect on bone regeneration among different LIPUS frequencies [40,43]. This discrepancy in conclusions may stem from differences in experimental models. For repairing superficial injuries, the damaged area may be more responsive to frequency variations, whereas for deep tissue repair, as energy attenuates, frequency difference may not be the primary influencing factor. This also suggests that the functional impact of different LIPUS frequencies on tissue repair warrants further in-depth investigation.

#### 2.2.2. Acoustic Intensity

The acoustic intensity of LIPUS (unit: W/cm^2^) reflects the energy carried by the acoustic wave per unit area. Typical LIPUS intensity is regulated to <3 W/cm^2^, far below the threshold for generating thermal ablation or stable cavitation. Therefore, its primary biological effect is a controllable acoustic-mechanical stimulation with minimal temperature rise. Studies have demonstrated that acoustic intensities within the low-intensity range of 30–100 mW/cm^2^ can promote stem cell proliferation and differentiation [44]. This result has also been corroborated by findings using adipose-derived mesenchymal stem cells (ASCs) laden within 3D piezoelectric hydrogels [45]. Regarding slightly higher intensities within the LIPUS range, studies indicate an association with the opening of mechanosensitive ion channels. At intensities around 1.0 W/cm^2^, LIPUS can transmit mechanical vibrations and activate cellular mechanosensitive ion channels such as TRPV1, TRPA1, and Piezo1, leading to the opening of calcium channels and subsequent ion influx [31,46]. (Figure 2b) Acoustic intensity is a crucial parameter in LIPUS. Excessively high intensity may lead to pronounced acoustic thermal effects, damaging surrounding tissues, while excessively low intensity may fail to elicit an effective biological response. Therefore, assessing and selecting an appropriate intensity is a key factor in ensuring both the safety and efficacy of the treatment.

#### 2.2.3. Duty Cycle

The duty cycle is defined as the ratio (%) of the “acoustic emission duration” to the total period time within a pulse cycle. It is a core parameter for controlling temperature rise, acoustic exposure dose, and pulse structure. At identical peak intensities, a higher duty cycle results in higher temporal-average acoustic energy. That is, within the same duration, the exposed part receives more frequent impacts of acoustic energy, leading to a slight temperature increase and enhanced mechanical effects. Conversely, a lower duty cycle maintains an intermittent mode of energy input, keeping the temperature rise process relatively stable. Therefore, the duty cycle is another key factor, besides acoustic intensity, that may influence the thermal characteristics of LIPUS. In practical applications, beyond thermal effects, the duty cycle has a non-negligible influence on the functional regulation of cells, such as the release of cytokines and the modulation of ion channels. Studies have shown that macrophages exposed to a 20% duty cycle exhibit a more pronounced decrease in pro-inflammatory cytokines compared to other parameters (e.g., 10%, 30%, 40%). Furthermore, two mechanosensitive ion channels, PIEZO1 and TRPV1, are involved in LIPUS-mediated regulation of cytokine release, accompanied by activation of the p38 MAPK pathway and inhibition of NF-κB signaling, all of which are associated with the duty cycle parameter [47]. Typically, when applying LIPUS, especially for optimizing the treatment of bone remodeling diseases, a 20% duty cycle is relatively commonly used [39]; however, literature also indicates that a higher duty cycle (50%) can promote the proliferation of Schwann cells and myelin formation [48] (Figure 2b). Therefore, for different applications, tissues, or disease stages, the selection of this parameter should be personalized and optimized to achieve better experimental or therapeutic outcomes.

#### 2.2.4. Stimulation Duration

Stimulation duration encompasses both the single-session treatment duration (e.g., 5–30 min) and the entire treatment cycle (e.g., continuously for 1–4 weeks). Since the biological effects of LIPUS stem from the cumulative mechanical signals rather than a one-time instantaneous energy exposure, both the treatment duration per session and the overall treatment cycle are often decisive for tissue repair [49]. It is generally believed that shorter, transient stimuli are often insufficient to activate adequate mechano-transduction signaling, while longer treatments can sustainably promote processes such as axonal regeneration, angiogenesis, and inflammation regulation. However, excessively long stimulation times may lead to a decline in cell viability. (Figure 2b) Nevertheless, the specific stimulation times and parameter combinations reported by different research groups vary considerably across experimental settings, reflecting substantial heterogeneity rather than a standardized framework tailored to distinct therapeutic purposes (e.g., promoting tissue repair in vivo versus enhancing cell differentiation in vitro). Uddin SM et al. found that relatively short-duration stimulation could promote normal osteogenic differentiation of mesenchymal stem cells [50]. Huang et al. revealed in their study that daily 5 min LIPUS stimulation effectively promoted the proliferation of hASCs, showing significant differences compared to the non-stimulated control group. However, daily 10 min stimulation led to a noticeable decrease in hASC viability. The experiment demonstrated that a regimen of 5 min per day, conducted continuously for a total of 4 days (with one stimulation session at 24, 48, 72, and 96 h of cell culture, respectively), resulted in a significant increase in the proportion of hASCs in the G1 and S phases (indicating accelerated cell cycle progression), upregulation of proliferation-related genes (CyclinD1, c-myc) and paracrine function gene (SDF-1α), along with reduced apoptosis rates, ultimately achieving effective proliferation [51]. In a study on sheep fracture healing, Brandon et al. employed a clinically validated fracture healing protocol of “20 min daily, five times per week,” using a 1.0 kHz repetition frequency and 30 mW/cm^2^ intensity output over a 4-month healing cycle. The results showed that the LIPUS group exhibited increased periosteal bridging, more mineralized tissue in the callus, and a significantly higher mineral apposition rate compared to the control group [52]. Therefore, similar to the selection of other parameters, to achieve optimal therapeutic outcomes, it is necessary to choose appropriate single-session treatment durations and treatment cycles tailored to the intended purpose.

The aforementioned key physical parameters of LIPUS (including frequency, acoustic intensity, duty cycle, and stimulation duration) and their general biological effects in different tissue types, along with the complexity of parameter configuration, underscore the significant importance of personalized medicine using LIPUS and lay the foundation for understanding the dose-effect relationship of ultrasound stimulation. However, compared to bone or soft tissues, the nervous system exhibits marked specificity in terms of structural hierarchy, signal conduction modes, and regeneration patterns post-injury. Consequently, identical LIPUS parameter combinations often induce more complex and hierarchical biological responses in neural tissues. In the following sections, the repair of neural tissues, specifically PNI and CNI, and their underlying mechanisms will be discussed in detail.

### 2.3. Key Biological Effects of LIPUS on Neural Repair–Associated Cells and Microenvironment

Although many early mechanistic insights into LIPUS were derived from bone, muscle, and mesenchymal stem cell systems, these studies primarily serve as foundational evidence for parameter-dependent mechano-transduction. The following sections will focus specifically on nerve-related applications. Neuronal cells and their supporting cells are highly sensitive to mechanical stimulation [53,54], and their functions are highly dependent on calcium signaling, cytoskeletal tension, and intercellular communication [55]. In this section, the roles of LIPUS in neural repair—specifically its regulation of calcium channels, cytoskeletal remodeling, cytokines and microenvironment improvement, inflammation modulation, and angiogenesis—will be discussed in detail.

**Figure 3:**
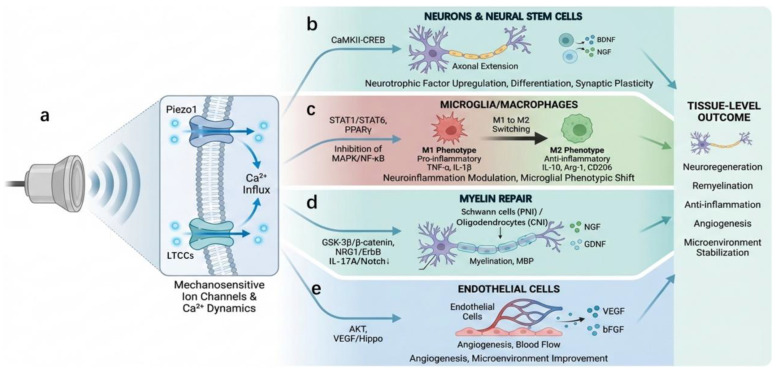
Multifaceted biological effects of LIPUS during nerve regeneration. Schematic illustration summarizing the key cellular and molecular responses elicited by LIPUS in neural repair. (**a**) LIPUS-induced activation of mechanosensitive and voltage-gated ion channels (e.g., Piezo1 and L-type Ca^2+^ channels), leading to rhythmic Ca^2+^ dynamics and downstream signaling. (**b**) Direct effects of LIPUS on neurons and neural stem/progenitor cells, including enhanced neuronal excitability, neurite outgrowth, differentiation, and neurotrophic factor secretion. (**c**) Modulation of neuroinflammatory responses by LIPUS through regulating microglial and macrophage polarization, characterized by suppression of pro-inflammatory phenotypes and promotion of anti-inflammatory states. (**d**) Activation of Schwann cells and oligodendrocytes by LIPUS, facilitating their proliferation, migration, neurotrophic support, maturation, and myelin-associated protein expression during peripheral nerve repair and central nerve repair. (**e**) Improvement of the regenerative microenvironment via LIPUS-mediated enhancement of endothelial cell activity and angiogenesis, thereby supporting metabolic supply and neurovascular coupling.

As illustrated in Figure 3, the biological effects of LIPUS can be conceptually organized into a hierarchical mechano-transduction cascade rather than a set of independent parallel pathways. Specifically, acoustic mechanical forces are first sensed by mechanosensitive membrane structures, which subsequently trigger calcium-dependent intracellular signaling and downstream transcriptional responses, ultimately leading to functional cellular outcomes.

#### 2.3.1. Activation of Mechanosensitive Ion Channels and Regulation of Ca^2+^ Dynamics

Owing to its inherent mechanical force-related characteristics, the biological effect of LIPUS in the nervous system involves the activation of mechanosensitive ion channels and the subsequent changes in Ca^2+^ dynamics. Ultrasound-induced periodic acoustic-mechanical stress can act on cell membrane-cytoskeleton linkage structures, activating mechanosensitive or voltage-dependent channels such as Piezo1 and L-type Ca^2+^ channels (LTCCs), leading to transient or periodic Ca^2+^ influx. (Figure 3a) Fan et al., using whole-cell patch-clamp recordings in their study, found that treating hippocampal neurons with 1 MHz LIPUS for 15 min increased the frequency and amplitude of spontaneous action potentials and spontaneous excitatory postsynaptic currents. Their research elucidated that LIPUS elevates intracellular calcium concentration by promoting the activation of LTCC, which further leads to the activation of the CaMKII-CREB pathway, thereby regulating gene transcription and protein expression [56]. Zhu et al. discovered in their study that the mechanosensitive ion channel Piezo1 is a primary mediator through which ultrasound exerts neuromodulatory effects, regulating neuronal signaling and animal behavior. They validated this by knocking out the Piezo1 protein (P1KO) in the right motor cortex of mice. Experiments revealed that this procedure significantly reduced ultrasound-induced neuronal calcium responses, limb movements, and electromyographic (EMG) responses. Furthermore, they found that Piezo1 protein expression levels were higher in the central amygdala (CEA), and the CEA was more sensitive to ultrasound stimulation than the cortex. Similarly, knocking out Piezo1 protein in CEA neurons significantly diminished the response to ultrasound stimulation [30]. Ca^2+^ surges in neural cells are not singular signals but act as a “second messenger hub” involved in regulating processes such as axonal extension, growth cone guidance, cell migration, and neural precursor cell differentiation. (Figure 3b) Unlike Ca^2+^ signals under injury or pathological conditions, those induced by LIPUS typically manifest as mild and rhythmic fluctuations, thereby providing an important basis for its safe application in neural regeneration.

#### 2.3.2. Secretion of Neurotrophic Factors

In addition to directly acting on neural cells, LIPUS can significantly regulate the paracrine signaling network associated with neural regeneration. This includes its combined use with relevant materials, such as piezoelectric materials, to modulate neural stem cells, induce their differentiation into neurons, and upregulate the expression of brain-derived neurotrophic factor (BDNF). Furthermore, multiple studies have shown that LIPUS stimulation with appropriate parameters can directly upregulate the expression of various neurotrophic and angiogenic factors, including BDNF, nerve growth factor (NGF), neurotrophin-3 (NT-3), and vascular endothelial growth factor (VEGF). Wang et al. developed piezoelectric nanostickers that can adhere to cell surfaces and generate microcurrents under ultrasound, promoting the differentiation of neural stem cells into neurons. They found that this piezoelectric material could release BDNF via the VGCC/Ca^2+^/CaMK II/CREB pathway [57]. (Figure 3b) Xia et al. indicated in their review that LIPUS can promote neurite outgrowth, enhance the expression of neurotrophic factors (such as BDNF and NGF), thereby facilitating neuroprotection and nerve repair/regeneration. Moreover, upregulated BDNF and NGF can enhance neuronal protective and repair functions, supporting neuronal survival, differentiation, and synaptic plasticity [58]. Wang et al. found that LIPUS stimulation alone was sufficient to promote rapid functional and histological improvement after sciatic nerve crush injury in rats and upregulate BDNF expression in their study on rat sciatic nerve crush repair [59]. This conclusion is consistent with the findings of the Liao team in a spinal cord injury repair model [60]. Their study revealed that neural stem cells regulate the microenvironment by secreting neurotrophic factors (such as BDNF and NGF), indicating that LIPUS can alter the pathological microenvironment by influencing neural stem cell function. Therefore, it is evident that these factors not only directly promote neuronal survival and axonal growth but also improve the regenerative microenvironment in the injured area by modulating neuron-glial cell interactions. Compared to single-agent drug supplementation, LIPUS-induced upregulation of endogenous factors offers spatial and temporal controllability, meeting the demand for dynamic regulation during the nerve repair process.

At the mechanistic level, the upregulation of neurotrophic factors such as BDNF and NGF is tightly linked to calcium-dependent signaling cascades [61,62]. As noted earlier, LIPUS can activate mechanosensitive ion channels and voltage-gated calcium channels, leading to an increase in intracellular Ca^2+^ concentration. This calcium influx in turn activates CaMKII and ERK pathways, culminating in CREB phosphorylation—a classic transcription factor known to regulate BDNF expression. Thus, although not all studies have explicitly resolved the specific contributions of individual channels, the observed upregulation of neurotrophic factors aligns mechanistically with Ca^2+^-dependent mechano-transduction, likely involving LTCCs and Piezo-mediated calcium entry.

#### 2.3.3. Regulation of Neuroinflammatory Responses

The inflammatory response following neural injury plays a positive role in the early stages by clearing debris and initiating repair. During the early inflammatory process, the early activity of monocytes/macrophages is crucial for effective remyelination, and their phagocytic clearance function is significant for removing toxic substances and inhibitory molecules that would otherwise hinder axonal regeneration and remyelination [63,64]. However, persistent or excessive inflammation can significantly inhibit regeneration. Macrophages exacerbate cell death and damage through the prolonged release of pro-inflammatory mediators, which is associated with inhibitory scar formation and poor regeneration [65]. Studies also indicate that excessive inflammation after the acute phase may impair axonal regeneration, leading to cell death and neural circuit degeneration [66]. Therefore, temporally controlled regulation of neuroinflammation at specific time points is highly meaningful. Accumulating evidence suggests that LIPUS can participate in regulating neuroinflammatory responses through its tunable mechanical stimulation parameters, particularly influencing the phenotypic switching of microglia and macrophages. Specifically, LIPUS can suppress the activation of pro-inflammatory M1-type microglia and reduce the expression of inflammatory cytokines such as TNF-α and IL-1β. Hsu et al. demonstrated in their study that LIPUS can attenuate the pro-inflammatory response in activated microglia and specifically prevent M1 polarization. The study found that LIPUS treatment significantly reduced the expression of inflammatory markers (e.g., iNOS, TNF-α, IL-1β, and IL-6) and M1 cell surface markers (e.g., CD86 and CD68) in LPS-stimulated microglia. Furthermore, the study proved that LIPUS treatment significantly enhanced the expression of M2-related markers (Arg-1, IL-10, and Ym1) and the membrane protein CD206. It was discovered that LIPUS inhibits microglial polarization and shifts microglia from a pro-inflammatory M1 phenotype to an anti-inflammatory M2 phenotype by modulating the STAT1/STAT6 and PPARγ signaling pathways [67]. Hsiau et al. also found that LIPUS can significantly reduce microglial activation and neuroinflammation, while decreasing M1 polarization and enhancing M2 polarization. Differently, they found this process was achieved through LIPUS stimulation, attenuating interferon-γ (IFN-γ)-induced microglial activation in vitro, inhibiting the release of pro-inflammatory cytokines, and suppressing the activation of the mitogen-activated protein kinase–nuclear factor-κB pathway [68]. Casagrande et al. developed curcumin–gold nanoparticles (Cur-AuNPs), which, when combined with LIPUS, can be used for neuromodulation in an Alzheimer’s disease model. This therapeutic approach can attenuate the phosphorylation of STAT1 and promote the phosphorylation of STAT6, thereby inducing a rapid shift from a pro-inflammatory M1 phenotype to a beneficial M2 phenotype and enhancing the release of beneficial mediators, including IL-10, CD206, and neurotrophic factors such as BDNF and NGF [69] (Figure 3c). Therefore, it is evident that although this process may involve different pathways, LIPUS is widely recognized as capable of modulating microglial activation to alleviate neuroinflammation, reduce oxidative stress, and enhance neuroprotection, thereby reducing neurotoxicity [58].

Currently, most studies on LIPUS-mediated microglial polarization have focused on the downstream validation of signaling pathways. However, accumulating evidence suggests that calcium-dependent mechano-transduction may represent an upstream regulatory layer. JF Espinosa-Parrilla et al. investigated the role of LTCCs in microglia and demonstrated that blockade of LTCCs with nifedipine reduced the release of pro-inflammatory factors such as TNF-α and nitric oxide from activated BV2 microglial cells, indicating that LTCCs are involved in the regulation of secretory activity [70]. Given that LTCCs can be activated by LIPUS, as discussed above, it is reasonable to speculate that calcium-dependent signaling contributes to the observed modulatory effects on microglial polarization. Nevertheless, direct validation targeting specific channels remains limited and warrants further investigation.

#### 2.3.4. Myelin Repair

In the repair process of peripheral nerve injury and central nerve injuries, myelin reconstruction is a critical determinant of functional recovery. It should be noted that Schwann cells are responsible for myelination in the peripheral nervous system, whereas myelin repair in the central nervous system is mediated primarily by oligodendrocytes. In the context of PNI, LIPUS has been proven to promote the proliferation and migration of Schwann cells. Li et al. found in their study that LIPUS could promote the proliferation, migration, and expression of the neurotrophic factor NGF in Schwann cells in vitro. In their established bilateral cavernous nerve injury model, they discovered that LIPUS enhanced Schwann cell-mediated neurite outgrowth from major pelvic ganglion neurons and explants [71]. Similarly, Ren et al. found that LIPUS could enhance the viability and proliferation capacity of Schwann cells, and increase the expression of growth factors and neurotrophic factors (including FGF, NGF, BDNF, and GDNF) secreted by Schwann cells. The underlying mechanism is that LIPUS promotes Schwann cell viability and proliferation by enhancing the GSK-3β/β-catenin signaling pathway [72]. Some studies also demonstrated that the effect of LIPUS on Schwann cell proliferation and myelination status may be mediated through the Neuregulin 1 (NRG1)/ErbB signaling pathway [73]. In the context of CNI, emerging evidence suggests that LIPUS can also modulate oligodendrocyte survival and maturation. Wang et al. demonstrated in their study that LIPUS stimulation protected oligodendrocytes and neurons, significantly upregulated the expression of myelin-associated proteins (such as myelin basic protein, MBP), enhanced myelin coverage, and improved early myelin loss by downregulating the interleukin-17A/Notch 1 signaling pathway [74]. Furthermore, Yang et al. reported that LIPUS could reduce demyelination in a rat model by accelerating oligodendrocyte maturation [75].

Studies have established that myelination is exquisitely sensitive to intracellular calcium dynamics. Both Schwann cells and oligodendrocyte precursor cells exhibit calcium-dependent regulation of proliferation, maturation, and myelin protein expression. Belfiore et al. demonstrated that LTCCs—particularly Cav1.2—participate in oligodendrocyte maturation and myelination, and reported that conditional knockout of Cav1.2 in mice leads to a reduction in myelinated axons, as well as decreased numbers of myelinating oligodendrocytes and proliferating OPCs [76]. In their review, Numata et al. further indicated that LTCCs facilitate Schwann cell proliferation and myelin remodeling via extracellular calcium influx, and that Piezo channels are capable of decoding mechanical stretch, thereby modulating neurotrophic factor secretion and myelination in Schwann cells [77]. Given that low-intensity pulsed ultrasound (LIPUS) has been demonstrated to activate mechanosensitive ion channels and evoke calcium influx in neural cells, it is reasonable to hypothesize that calcium-dependent signaling may function as an upstream integrative mechanism bridging mechanical stimulation with glial activation and remyelination.

#### 2.3.5. Activation of Angiogenesis

The regeneration of neural tissue is highly dependent on adequate blood supply and metabolic support. Neural tissue regeneration is a highly energy-consuming process that involves axonal regeneration, myelination, and cell proliferation. A nascent vascular network can deliver oxygen and glucose while removing metabolic waste, thereby providing a more stable internal microenvironment for nerve repair. Furthermore, studies have shown that endothelial cell–conditioned medium can significantly enhance the neurotrophic effects of conditioned medium derived from induced multipotent progenitor cells, indicating a synergistic interaction between endothelial and progenitor cell–mediated signaling. The potential of multipotent progenitor cells to promote nerve growth is achieved through VEGF-A-dependent actions, while endothelial cells can also secrete BDNF, supporting the survival and integration of newly recruited neurons [78,79] (Figure 3e). Currently, there is no extensive research conclusively proving that LIPUS can promote nerve repair by inducing angiogenesis after neural injury. However, there are numerous examples demonstrating LIPUS’s ability to promote angiogenesis. For instance, Li et al. found in their study that LIPUS stimulates angiogenesis by promoting the production of related cytokines such as IL-8, bFGF, and VEGF, and can promote angiogenesis via the AKT and VEGF/Hippo signaling pathways [80]. Similarly, Ji et al. demonstrated in their study that LIPUS treatment promotes angiogenesis, significantly improving capillary density and myocardial blood flow in a porcine model of ischemic cardiomyopathy. This pathway is also achieved by stimulating endothelial cells to release VEGF and bFGF. In addition, accumulating evidence indicates that LIPUS promotes endothelial cell proliferation, which may facilitate endothelial cell–mediated neurotrophic support. For instance, Shindo et al. reported that LIPUS markedly enhanced the proliferative capacity of HUVECs and upregulated the expression of angiogenesis-related factors, including VEGF and endothelial nitric oxide synthase (eNOS). Mechanistically, these effects were mediated through activation of the β1-integrin/caveolin-1/FAK/ERK/Akt signaling pathway, thereby promoting endothelial cell proliferation and angiogenesis [81]. These findings are highly consistent with those reported by Eguchi et al. in studies focusing on vascular dementia and Alzheimer’s disease, which further support the pro-angiogenic and vasoprotective roles of LIPUS. In combination with biomaterials, Kang et al. employed LIPUS to stimulate human adipose-derived stem cells (hADSCs) and human umbilical vein endothelial cells (HUVECs) cultured on collagen/hyaluronan scaffolds. Their results demonstrated that LIPUS significantly enhanced the proliferation of both cell types, accompanied by a marked upregulation of angiogenesis-related markers, including CD31 and vascular endothelial cadherin mRNA. These findings indicate that LIPUS possesses the capacity to promote endothelial cell proliferation and angiogenic activity [82]. Given the strong dependence of neural tissue regeneration on angiogenesis and the promotive effects of LIPUS on this process, it is reasonable to postulate that in neural injury models, LIPUS may promote the stabilization of the injured internal microenvironment through the promotion of angiogenesis and improvement of blood flow, and upregulate the secretion of nerve repair-promoting factors by endothelial cells.

Mechanistically, endothelial cells are highly sensitive to mechanical stimuli and express calcium-permeable ion channels, including Piezo1. Chen et al. demonstrated that Piezo1 senses mechanical stimuli and mediates various biological processes, such as angiogenesis. Deletion of endothelial Piezo1 in mice leads to downregulation of calcium-activated proteolytic calpain activity during vascularization [83]. Activation of these channels has been shown to regulate intracellular calcium dynamics, thereby influencing the expression of vascular endothelial growth factor (VEGF), Akt signaling, and endothelial nitric oxide synthase (eNOS) activity [84]. Therefore, considering the aforementioned role of LIPUS in activating Piezo1 channels, it is reasonable to infer its functional contribution to angiogenesis during nerve repair.

Collectively, these mechano-transduction pathways and parameter-dependent characteristics establish a general biophysical framework through which LIPUS interacts with biological systems, and these principles provide the foundational basis for understanding its application in specific tissues. Against this mechanistic background, increasing attention has been directed toward the role of LIPUS in neural repair.

## 3. Application of LIPUS in the Central and Peripheral Nerve System

Building upon these general mechano-transduction principles and parameter-dependent effects, increasing attention has been directed toward the application of LIPUS in nervous system repair.

**Figure 4:**
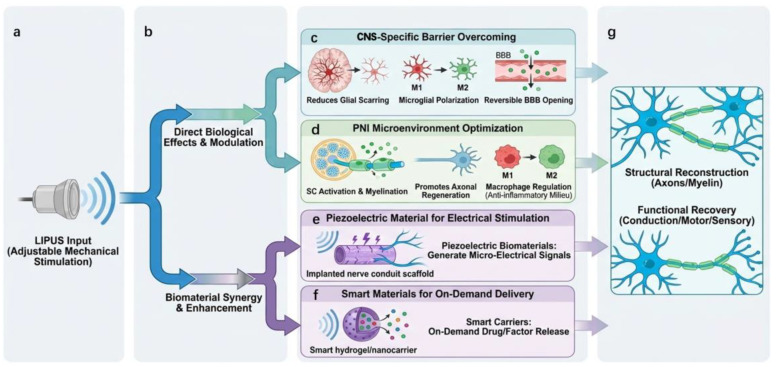
Core strategies and mechanisms of LIPUS in central and peripheral nerve repair. (**a**) LIPUS serves as an adjustable source of mechanical stimulation for both the CNS and PNI. (**b**) LIPUS-mediated neuromodulation proceeds through two major pathways: direct biological regulation and biomaterial-assisted enhancement. (**c**,**d**) Representative mechanisms by which LIPUS modulates pathological barriers and regenerative processes in CNI and PNI, respectively. (**e**,**f**) LIPUS-triggered activation of piezoelectric biomaterials and on-demand release from smart hydrogels or nanocarriers to amplify regenerative cues. (**g**) Integrated outcomes of LIPUS-based therapies, culminating in structural reconstruction and functional recovery.

Nerve injuries encompass central nerve injury (CNI), such as spinal cord injury, white matter injury, and traumatic brain injury (TBI), as well as peripheral nerve injury (PNI), such as sciatic nerve transection or compression. These injuries often lead to severe motor, sensory, and even autonomic functional loss. Peripheral and central nerve injuries differ in their intrinsic regenerative capacity, structural organization, and post-injury microenvironment. In the PNS, axonal regeneration is supported by Schwann cells, which rapidly proliferate, clear myelin debris, secrete neurotrophic factors, and form bands of Büngner. Peripheral nerves are capable of spontaneous regeneration over limited distances; therefore, from an engineering perspective, PNS repair primarily involves modulating the existing regenerative environment. In contrast, the regenerative capacity of the CNS is considerably more limited. Following injury, reactive astrocytes, microglia, and fibroblast-like cells contribute to the formation of fibrotic scars and glial scars, which pose both physical and molecular barriers to axonal regeneration. Additionally, the extracellular matrix contains chondroitin sulfate proteoglycans (CSPGs), which further inhibit axonal outgrowth. Therefore, repair of the central nervous system requires not only stimulation of intrinsic neuronal growth potential but also modulation of the inhibitory microenvironment. Their high treatment difficulty and limited regenerative potential make them significant clinical challenges. As a non-invasive, parametrically adjustable physical therapeutic strategy, LIPUS offers a novel approach to nerve repair through mechanical stimulation and downstream biological effects. (Figure 4a) As previously mentioned, LIPUS plays several specific roles in the nerve injury repair process, such as activating ion channels and neurotrophic factors, thereby promoting functional and structural reconstruction. Its efficacy has been corroborated in multiple studies. For instance, it significantly enhances axonal regeneration and neurotrophic factor expression in a sciatic nerve crush model [59]. In a sciatic nerve autograft model, LIPUS of different intensities improved healing rates and histological indicators [85]. Therefore, this chapter focuses on the applications of LIPUS in central and peripheral nerve injury repair, while also addressing its roles in regulating neurovascular barriers, with an emphasis on biological effects, mechanistic pathways, and therapeutic outcomes across different neural contexts. (Figure 4b).

### 3.1. Biological Effects and Mechanisms of LIPUS in Peripheral Nerve Injury (PNI)

LIPUS demonstrates significant potential for tissue reconstruction and functional recovery in PNI repair. (Figure 4d) Its mechanisms of action involve cellular-level signal regulation, modulation of inflammatory responses, and direct effects on axonal/myelin regenerative components. A substantial body of pre-clinical research consistently indicates that repeated LIPUS interventions can promote nerve fiber regeneration, myelin thickening, axonal extension, and the recovery of functional conduction velocity, without significant negative effects. The emergence of these effects relies on the multi-target regulatory mechanisms of LIPUS on the peripheral nerve microenvironment [86].

Schwann cells are the core supportive cells in PNI repair, and their proliferation, migration, and pro-myelinating capabilities directly determine the efficiency of axonal regeneration [87]. LIPUS can stimulate Schwann cells via mechanical signals, significantly enhancing their growth activity and secretion of neurotrophic factors. In earlier work, Zhang et al. investigated the effects of LIPUS on the proliferation of rat Schwann cells and NT-3 mRNA expression. Using parameters of 1 MHz ultrasound frequency, a spatial average intensity of 100 mW/cm^2^, applied for 5 min daily, they found that compared to the control group, NT-3 mRNA expression in the experimental group was significantly upregulated 14 days after LIPUS stimulation [88]. PNI is typically accompanied by a pronounced inflammatory response. Fontana et al. treated Schwann cells with H_2_O_2_ and evaluated the effect of LIPUS. Their study showed that parameters of 5 MHz and 500 mW cm^−2^ increased the release of neurotrophic cytokines (including nerve growth factor, brain-derived neurotrophic factor, and glial cell line-derived neurotrophic factor) in RSC96 cells when compared with the control group [89]. LIPUS could also act on the GSK-3β/β-catenin signaling pathway, aiding Schwann cells in entering a proliferative state and secreting a molecular environment supportive of regeneration [72,90]. Furthermore, research on pathways related to Schwann cell myelination showed that LIPUS can increase the expression of Neuregulin 1 (NRG1) and its receptors ErbB2 and ErbB3, enhancing signaling through the NRG1/ErbB axis. This suggests LIPUS not only promotes cell proliferation but also enhances their pro-myelinating properties, thereby improving the myelin thickness and functional recovery potential of regenerating nerves [73].

Multiple studies indicate that LIPUS can promote axonal regeneration and guidance, thereby improving functional indicators such as nerve conduction velocity (NCV) and muscle re-innervation. These improvements are associated with myelin thickening and re-myelination, suggesting LIPUS may promote the re-differentiation of repair-type Schwann cells into myelinating Schwann cells, thus enhancing nerve function. Concurrently, LIPUS can increase the number, diameter, or degree of myelination of axons distal to the injury site, indicating its role in promoting axonal growth and repair [91]. Peng et al. reported that repeated LIPUS significantly promoted axonal regeneration, increased the number and myelination of axons, improved NCV after nerve injury, and significantly promoted axonal regeneration and muscle reinnervation [86]. In a study by Jiang et al., LIPUS intervention significantly accelerated the speed and quantity of axonal regeneration post-injury in a rat sciatic nerve autograft model, suggesting ultrasonic mechanical stimulation can directly influence the axonal structural reconstruction process [85].

Moreover, early after PNI, a complex inflammatory response is typically triggered. The inflammatory cascade can both promote debris clearance and cause secondary damage. LIPUS has been found to possess the ability to modulate pro- and anti-inflammatory mediators, helping to create a neural microenvironment more conducive to regeneration. Studies showed that LIPUS could significantly inhibit the mature form of IL-1β both in vitro and in vivo and increase macrophage autophagy levels, further supporting its role in regulating post-PNI inflammation. LIPUS could also degrade PKM2 via an SQSTM1-dependent autophagic pathway, thereby inhibiting the mature release of IL-1β, a mechanism validated in inflammation models [92]. Simultaneously, LIPUS can downregulate pro-inflammatory factors such as TNF-α and IL-6, and inhibit the expression of nerve growth inhibitors SEMA3A and GSK3β, thereby improving the regenerative microenvironment [93]. At the cellular regulation level, LIPUS, similar to other physical therapies (e.g., low-level laser therapy), can promote macrophage polarization towards the M2 phenotype and enhance the secretion of neurotrophic factors by activating the PKA-CREB pathway, further supporting axonal regeneration [94]. Additionally, LIPUS might accelerate myelin debris clearance by regulating Schwann cell autophagy, a process involving the p75NTR/AMPK/mTOR signaling axis, thereby promoting structural repair and functional recovery in the early stages of injury [95]. Therefore, LIPUS creates a favorable local microenvironment for neural regeneration after PNI through synergistic regulation of inflammatory responses, cellular autophagy, and neurotrophic factor secretion.

Beyond providing indirect support via Schwann cells, evidence indicates that LIPUS can directly stimulate neurons and their axons, enhancing neurite growth. LIPUS can promote neurite growth and nerve regeneration by activating the Netrin-1/DCC signaling pathway, or directly activate neurons via Acid-Sensing Ion Channel-1a [96]. Similarly, LIPUS can also enhance neurotrophic factor-induced neurite growth by activating mechano-transduction-mediated signaling pathways (e.g., ERK1/2-CREB-Trx-1) [97,98]. Pi et al. developed piezoelectric nanotracts that can work synergistically with LIPUS to jointly increase neurite growth length and the percentage of PC12 cells containing neurites.

In summary, based on the existing literature, the biological effects of LIPUS in PNI repair involve multiple key mechanisms, including Schwann cell activation and proliferation, enhanced myelin reconstruction, promotion of axonal regeneration, regulation of neurotrophic factors, suppression of inflammatory responses, and direct action on neuronal signaling pathways. These effects not only structurally improve the regenerative state of the injured nerve but are ultimately reflected in functional improvements such as nerve conduction velocity and sensory/motor functional recovery. Future efforts to further define the molecular networks and parameter-dependent characteristics of these actions will provide more specific guidance and optimization strategies for the clinical translation of LIPUS.

### 3.2. Biological Effects and Mechanisms of LIPUS in Central Nerve Injury (CNI)

Although CNI and PNI exhibit significant differences in anatomical structure, regenerative capacity, and immune microenvironment, they still share certain commonalities in their biological responses to LIPUS. For instance, LIPUS-mediated mechano-transduction, Ca^2+^ signaling regulation, alleviation of inflammatory responses, and direct stimulation of neurons and axonal growth have been widely reported in both PNI and CNI. Therefore, this section will not reiterate these universal mechanisms but will instead focus on the biological barriers unique to CNI and the distinctive role of LIPUS in overcoming them, including glial scarring, the inhibitory molecular environment, the blood–brain barrier, and the complexity of central immune responses (Figure 4c).

Glial scarring is a core pathological feature contributing to regeneration failure in CNI. After injury, astrocytes undergo reactive activation characterized by hypertrophy and spatial rearrangement, associated with strong upregulation of the intermediate filament protein (GFAP, nestin and vimentin), forming a dense astrocyte-rich border zone commonly referred to as the glial scar [99]. This structure primarily functions as a molecular inhibitory barrier, largely due to the excessively deposited chondroitin sulfate proteoglycans (CSPGs), which severely inhibit axonal regeneration [100]. The physical obstruction that directly blocks axonal penetration mainly arises from the fibrotic scar located within the lesion core, which is enriched in extracellular matrix (Type I collagen, fibronectin, etc.) produced by fibroblast-like cells. Increasing evidence suggests that LIPUS predominantly modulates reactive astrocyte activation and glial scar–associated molecular inhibition, rather than directly degrading the fibrotic scar [99]. Specifically, Su et al. constructed a mouse model of intracerebral hemorrhage. After applying LIPUS stimulation, the levels of astrocyte activation and reactive astrocytes were reduced. This process diminished microglial activation within reactive astrocytes and the resulting neurotoxicity, further attenuating the formation of the glial scar [101]. Liao et al. also found in their study that, compared to other groups in vivo after spinal cord injury (SCI), the group treated with NSCs/LIPUS+ showed a reduction in the astrocytic marker GFAP, suggesting suppressed glial cell differentiation and glial scar formation [60]. Li et al., focusing on intracortical brain–computer interface research, found that electrode implantation into the cerebral cortex activates microglia, triggering an inflammatory cascade. LIPUS can reduce microglia-mediated neuroinflammation following microelectrode implantation. Data showed that LIPUS stimulation improved tissue healing and surveillance functions, decreased microglial coverage of the microelectrodes, and reduced astrocytic glial scarring. The results indicate that LIPUS helps diminish the foreign body response around chronic intracortical microelectrodes [35]. Unlike the “supportive Schwann cell-dominated” repair mode in PNI, a key role of LIPUS in CNI lies in limiting excessive glial reactions to create a favorable repair microenvironment for axonal regeneration.

Unlike the inflammatory response in PNI, which is dominated by peripheral macrophages, inflammation in CNI is primarily driven by microglia and infiltrating monocytes. LIPUS has been confirmed to significantly inhibit the overactivation of microglia. Hsiau et al. found that LIPUS attenuates interferon-γ-induced microglial activation in vitro by inhibiting the mitogen-activated protein kinase–nuclear factor-κB (MAPK-NF-κB) signaling pathway, and suppresses the release of nitric oxide (NO) and pro-inflammatory cytokines. This led to reduced M1 microglial polarization, enhanced M2 polarization, and promoted a reparative microglial phenotype [68]. This process was similarly validated in the study by Hsu’s team [67]. Su et al. demonstrated in their study that under experimental intracerebral hemorrhage conditions, LIPUS treatment reduced COX-2 levels in primary astrocytes and thrombin-induced cells. Its protective effect relies on inhibiting PI3K/Akt-NF-κB signaling to reduce glia-mediated inflammation [101]. It is noteworthy that in CNI, the immunomodulatory effects of LIPUS are not only related to regeneration but are also closely associated with neuroprotection and the suppression of secondary injury.

Overall, the role of LIPUS in CNI extends beyond merely “promoting regeneration” to more prominently reshaping the inhibitory central microenvironment and limiting secondary damage. It is precisely these CNI-specific biological barriers that position LIPUS as an important hub connecting physical stimulation, biomaterials, and precise delivery strategies, laying the groundwork for its subsequent deep integration with intelligent materials and AI parameter optimization.

### 3.3. LIPUS-Enabled Modulation of Neurovascular Barriers for CNS Drug Delivery

The blood–brain barrier (BBB) and blood–spinal cord barrier (BSCB) represent major physiological obstacles limiting the delivery of therapeutic agents to the central nervous system (CNS). Recent studies have shown that within safe parameter ranges, LIPUS (especially when combined with microbubbles) can achieve reversible BBB and BSCB opening, thereby enhancing the delivery efficiency of drugs, biomacromolecules, and nanocarriers to the injury site without causing permanent structural damage. Li et al. used Evans blue staining to demonstrate that specific combinations of ultrasound intensity (0.6, 0.8, and 1.0 W/cm^2^) and duration (1, 3, and 5 min) could sufficiently open the BBB, indicating LIPUS as a promising technology for enhancing targeted drug delivery [102]. Similarly, Luo et al. also found that LIPUS can temporarily, repeatedly, locally, and sustainably open the BBB. By employing LIPUS stimulation, they enhanced the delivery of 30 nm coenzyme Q10 nanoparticles to the brains of an Alzheimer’s disease (AD) mouse model [103]. Also, in an AD model, Casagrande et al. used LIPUS, which induced temporary BBB opening, promoting the delivery of curcumin–gold nanoparticles (CUR-AuNPs) to the brain. Liu et al. employed an ultrasound-mediated nanosystem (Qc@SNPs-MB combined with LIPUS) for drug delivery to brain tissue. The results proved that this system significantly improved cognitive levels, reduced amyloid-β content, and inhibited neuronal loss in AD mice, demonstrating therapeutic effects for central nervous system diseases [69,104]. Collectively, these findings suggest that LIPUS functions not only as a neuromodulatory stimulus but also as a spatiotemporally controllable tool for enhancing CNS drug delivery by transiently modulating neurovascular barriers.

Table 2 summarizes the key experimental parameters used in the aforementioned major studies. Although the parameters vary across different studies, discernible patterns can still be identified, and the findings remain consistent with conclusions drawn previously. In the field of neural stimulation, a relatively low frequency is generally required to ensure greater penetration depth. As mentioned earlier, high intensity often carries the risk of thermal damage or necrosis. In neural repair studies, in order to achieve functional recovery and induce cell division and differentiation by LIPUS stimulation, the intensities employed in experiments are usually below 1 W/cm^2^.

Comparative analysis reveals that, consistent with earlier conclusions, promoting cell proliferation and differentiation via LIPUS generally involves the use of lower intensities, often less than 100 mW/cm^2^. Studies on Schwann cells preliminarily suggest that a relatively high duty cycle (e.g., 50%) tends to be more suitable for enhancing cell proliferation. At the functional recovery level, comparison of the data in the table indicates that LIPUS stimulation with intensities above 100 mW/cm^2^ usually plays a significant role in functional restoration, such as functional reconstruction, axon regeneration, increased myelinated nerve fiber density, upregulation of neural markers like BDNF and NGF, and modulation of the microenvironment.

Therefore, by synthesizing the correspondence between the above parameters and the functional outcomes of LIPUS in neural repair, we conclude that for neural repair research, especially in vivo studies and applications, 1 MHz is generally an appropriate frequency [57,75,85,87,88,93,94]. When aiming to promote cell proliferation and differentiation, a higher duty cycle (e.g., 50%) combined with a lower intensity (typically below 100 mW/cm^2^) may be adopted. In contrast, for stages focused on functional reconstruction, a duty cycle around 20%, along with a relatively higher intensity (0.1–1 W/cm^2^), is likely a more suitable choice.

## 4. Synergy of LIPUS with Biomaterials: Strategies and Progress

Although LIPUS demonstrates significant advantages in regulating cellular mechano-sensation, inflammatory responses, and nerve regeneration-related signaling pathways, the effect of a single physical stimulus within the complex microenvironment of neurological diseases remains limited. For example, for CNI and PNI, the neural injury site is often accompanied by structural loss, insufficient nutritional support, hypoxia, and the persistent presence of inhibitory molecules. Relying solely on LIPUS makes it difficult to maintain a favorable regenerative state over the long term. Therefore, recent research has gradually shifted from “LIPUS as an independent treatment” to “LIPUS as a synergistic regulation tool,” aiming to amplify its therapeutic effects spatially, temporally, and biologically through combination with biomaterials. Apart from supporting scaffold, as mentioned earlier, LIPUS can serve as a means to temporarily open the BBB and BSCB, enabling biomaterials such as nanoparticles to act as drug delivery systems for precise drug transport to the lesion site [105,106]. Beyond LIPUS-mediated modulation of neurovascular barriers, alternative drug delivery approaches, including mechanosensitive materials, have likewise been explored for the treatment of neurological diseases. Thus, there are numerous similar strategies, which not only help improve the local regenerative microenvironment but also provide a material basis for the precision and sustainability of LIPUS’s effects.

### 4.1. Piezoelectric Biomaterials for LIPUS-Driven Electrical Stimulation

In the field of neural injury repair, the core functions of nerve conduits and scaffold materials are to bridge the defect and provide a physical path for axonal regeneration. However, traditional conduits primarily offer static structural support and are difficult to actively regulate the complex regenerative microenvironment [107]. In recent years, combining LIPUS with nerve conduits has provided an innovative strategy for constructing an active regulation platform. LIPUS-driven piezoelectric conduits represent a broader electromechanical coupling strategy in nerve regeneration. By converting acoustic mechanical energy into localized electrical signals, piezoelectric biomaterials enable wireless and spatially confined electrical stimulation without the need for implanted electrodes or external power sources. This strategy relies on activating implanted piezoelectric biomaterials to generate a localized, controllable electric field, thereby mimicking endogenous bioelectrical signals to directly promote Schwann cell maturation, myelination, and directional extension of neuronal axons [108,109,110,111] (Figure 4e). Mechanistically, the electric fields generated by these materials are closely associated with calcium-dependent signaling. Microcurrent stimulation may activate voltage-gated calcium channels, modulate intracellular Ca^2+^ dynamics, and subsequently regulate downstream pathways involved in Schwann cell maturation, axonal elongation, and myelin formation. For example, Jeon et al. developed a piezoelectric nerve conduit (APNF-NGC). This conduit is fabricated via electrospinning using a blend of poly(L-lactic acid) and polyethylene glycol. Under LIPUS irradiation, it can generate an axially oriented electric field, effectively guiding directional axonal extension and Schwann cell migration. The electric field stimulation can activate voltage-gated calcium channels in neurons, inducing calcium influx, which in turn activates downstream signaling pathways (e.g., cAMP/PKA), upregulates growth-associated proteins (e.g., GAP-43), and promotes axonal regeneration. The electric field can also enhance the expression of neurotrophic factors (e.g., NGF, BDNF), further supporting nerve repair and myelination [108]. Similarly, Chen et al. developed a biodegradable piezoelectric nerve conduit using polycaprolactone and β-glycine as materials in their study. Although this study did not employ LIPUS as the mechanical force source, it similarly relied on the microcurrent generated by the material to achieve cellular-level stimulation. The results showed that the microcurrent generated by the piezoelectric material under stimulation could achieve a significant increase in myelin length and formation rate, as well as a significant increase in neurite length and differentiation ratio, resulting in more orderly alignment [109]. Table 3 summarizes several piezoelectric materials used for neural repair and their corresponding biological effects, including but not limited to ultrasound-triggered microcurrent generation, thereby providing a more intuitive functional comparison.

### 4.2. LIPUS-Responsive Biomaterials for On-Demand Therapeutic Delivery

Going a step further, many researchers are extending the effects of LIPUS from simple electrical stimulation to spatiotemporally controllable drug delivery by constructing multifunctional composite conduits. (Figure 4f) Some hydrogels, microspheres, or composite materials incorporate mechanically sensitive structures that can undergo conformational changes or mechanical dissociation under LIPUS, thereby releasing encapsulated neurotrophic factors or drugs. This approach makes the release of bioactive factors no longer dependent on passive diffusion but precisely regulated by exogenous physical signals, significantly improving spatiotemporal resolution. For instance, in the study by Xu et al., a thermosensitive hydrogel (pNIPAM hybrid hydrogel) loaded with nerve growth factor (NGF) was combined with piezoelectric nanofibers. While LIPUS triggered the piezoelectric electric field, its heat-generating effect also induced hydrogel contraction, enabling on-demand NGF release, thus simultaneously providing electrical stimulation and neurotrophic support [110]. Similarly, Yi et al. used electrospinning technology to prepare a bifunctional layered nerve conduit loaded with NGF and exhibiting high piezoelectric responsiveness. Heparin-functionalized chitosan nanofibers were used to load NGF, combining it with piezoelectric materials to promote peripheral nerve regeneration. Gopal’s article also employed the combination of LIPUS with materials for drug release. The researchers pointed out that the mechanical waves of LIPUS cause microscopic motion and rearrangement of the nanofibers, instantane\ously increasing the porosity of the fiber network and shortening the drug diffusion path. The generated electric field weakens the interaction between the hydrophobic drug retinoic acid (RA) and the polymer matrix (PLA/SF), promoting RA desorption from the fibers and accelerating its diffusion into the surrounding medium, achieving on-demand pulsed release [119]. Therefore, the combination of LIPUS with intelligent biomaterials has successfully upgraded nerve conduits from passive support structures to intelligent materials capable of responding to external commands and dynamically regulating the local electrical and chemical microenvironment. These strategies help enhance local biological effects within critical time windows while reducing systemic side effects, providing a possibility for constructing an on-demand release therapeutic system. (Figure 4g).

## 5. Outlook and Challenges

In recent years, fields such as artificial intelligence (AI) and machine learning (ML) have undergone rapid development. The life sciences are no longer solely dependent on traditional in vivo and in vitro experimental methodologies for efficacy validation, mechanism exploration, and material/drug selection. As previously discussed, combining LIPUS with piezoelectric nerve conduits, conductive scaffolds, or controllable drug-release materials has significantly expanded the functional boundaries of ultrasound in neural repair. This enables it not only to directly regulate cell behavior but also to remotely trigger electrical stimulation or the release of biological factors. Such composite systems often involve multi-parameter coupling, encompassing material property parameters, ultrasound stimulation parameters, and biological feedback. The therapeutic outcome is not determined by a single factor but depends on the combination of multiple parameters and the timing of stimulation. Against this backdrop, ML and AI provide a new technological pathway for constructing a “predictable, optimizable, and personalized” LIPUS-material neural repair system.

Specifically, within the synergistic system of LIPUS and biomaterials, ultrasound parameters (e.g., frequency, intensity, duty cycle) not only determine the intensity of mechanical stimulation within the tissue but also directly influence the amplitude of the electric field generated by piezoelectric conduits or the rate of drug release. ML models can integrate experimental data to establish a mapping relationship among LIPUS parameters, the physical response of the materials, and the biological effects on cells/tissues. For example, based on regression models or deep learning networks, it is possible to predict the electrical output level of a piezoelectric nerve conduit under different parameter combinations and its promoting effect on axonal growth or Schwann cell myelination.

Furthermore, AI has the potential to advance LIPUS-based neural repair into a stage of personalization and intelligence. By integrating multimodal data, such as electrophysiological functional assessment results and molecular or transcriptomic data, AI models can predict therapeutic responses under different injury types, injury stages, and even individual variations. For instance, the differing cell fates of neural stem cells and neurons during the early, middle, and late stages of spinal cord injury directly impact the choice of treatment modality for each stage. Predictions made by AI models can guide the selection of the appropriate LIPUS-triggered functional mechanism, such as employing microcurrent stimulation or activating a drug release system, thereby maximizing therapeutic efficacy while ensuring safety.

Beyond interdisciplinary integration, the current development and application of intelligent biomaterials offer new perspectives for treating various diseases. The adoption of multimodal synergistic stimulation may provide additional assistance in treating neural injuries. A single physical stimulus often struggles to comprehensively regulate the complex pathological microenvironment following CNI/PNI. Combining LIPUS with other external physical fields, such as light, magnetic fields, or electricity, holds promise for achieving fine-tuned spatiotemporal control over neuroinflammation, axonal regeneration, and neural circuit reconstruction. For example, the synergistic application of electroactive or piezoelectric materials with magnetically responsive nanosystems can enhance stimulation specificity and controllability while maintaining non-invasiveness. However, the integration of multimodal systems also significantly increases engineering complexity, imposing higher demands on device consistency, safety, and mechanistic understanding.

Finally, the transition from basic research to clinical translation still faces significant obstacles. Currently, most research remains at the stage of small animal models, with a lack of unified standards for parameters, models, and evaluation metrics across different experimental systems. This leads to insufficient comparability and reproducibility of research findings. Additionally, the acoustic windows for the central nervous system are limited by structures such as the skull and spine. How to achieve stable and precise energy delivery while ensuring safety is a practical issue that urgently needs resolution for the clinical application of LIPUS. In the future, combining AI-driven parameter optimization, multimodal physical stimulation strategies, and scalable, standardized instrumentation platforms may provide new breakthroughs for the precision, personalization, and clinical feasibility of LIPUS in neural injury repair.

## 6. Conclusions

LIPUS, as a non-invasive and adjustable physical therapy modality, demonstrates broad prospects in the treatment of neurological diseases. This article comprehensively elaborates on the biological basis of LIPUS in promoting neural repair at the cellular and microenvironmental levels, including its action systems, key parameters, and multiple mechanisms such as mechano-transduction, calcium signaling modulation, secretion of neurotrophic factors, inflammation regulation, and angiogenesis.

In PNI, LIPUS improves functional recovery by activating Schwann cells, promoting axonal regeneration, and facilitating myelin remodeling. In CNI, its effects focus more on modulating microglial polarization and inhibiting glial scar formation. Particularly noteworthy is the combination of LIPUS with intelligent biomaterials (such as piezoelectric scaffolds and LIPUS-responsive drug carriers), which has expanded its function from mere mechanical stimulation to a synergistic regulatory platform integrating electrical stimulation and on-demand drug release, significantly improving spatiotemporal precision and biological efficacy.

Despite its great potential, the clinical translation of LIPUS still faces challenges such as parameter standardization, mechanistic consistency, and accurate energy delivery to deep tissues. In the future, integrating artificial intelligence-driven parameter optimization, multimodal physical stimulation strategies, and standardized device platforms is expected to advance LIPUS toward intelligent, personalized, and clinically practical applications, providing novel solutions for the treatment of neurological diseases.

## Figures and Tables

**Table 1:** Summary of typical parameters and biomedical applications of high-intensity focused ultrasound (HIFU) and low-intensity pulsed ultrasound (LIPUS).

Parameter	LIPUS [25,26,27]	HIFU [28,29]
Typical frequency	1–3 MHz	0.2–7 MHz (often focused)
Spatial-average intensity	Commonly ≤3 W/cm^2^	>5 W/cm^2^ (typically 100–10,000 W/cm^2^)
Duty cycle	Pulsed	Often high duty
Thermal effect	Minimal (<1–2 °C rise)	Rapid temperature elevation (>60 °C)
Dominant mechanism	Mechanical stimulation, mechano-transduction, and ion channel activation	Thermal coagulative necrosis, cavitation
Cavitation	Generally avoided	Can be induced intentionally
Primary application	Regeneration, neuromodulation, and mechanobiology	Tumor ablation, tissue destruction

**Table 2:** Key experimental parameters and function outcomes of LIPUS stimulation in different CNS and PNS repair studies.

Target Tissue/Model	Frequency (MHz)	Acoustic Intensity (mw/cm^2^ or w/cm^2^)	Duty Cycle (%)	Stimulation Duration	Main Biological/Functional Outcomes	Reference
Rat Schwann cells (in vitro)	1.5 MHz	20 mW/cm^2^	50%	10 min/day for up to 7 days	Schwann cell proliferation upregulation; Krox20 and MBP expression upregulation; NRG1, ErbB2, ErbB3 expression upregulation	[66]
Rat Schwann cells (in vitro)	1 MHz	100 mW/cm^2^	30%	5 min/day	NT-3 mRNA expression upregulation; Schwann cell proliferation upregulation; BDNF mRNA expression downregulation	[77]
Rat Schwann cells (in vitro)	1 MHz	27.5 mW/cm^2^	-	10 min/day for 5 days	Increased cell viability and cell proliferation; FGF, NGF mRNA and protein expression upregulation	[65]
Rat sciatic nerve crush injury model (PNS)	1 MHz	140 mW/cm^2^	20%	5 min/day, started on day 1 after surgery, daily until day 14, then 5 days/week until sacrifice	Function restoration; axon regeneration; myelinated nerve fiber density upregulation; BDNF gene and protein expression upregulation	[56]
Rat sciatic nerve crush injury model (PNS)	1 MHz	140 mW/cm^2^	20%	5 min/day, 5 days/week	Downregulation of TNF, IL-6, SEMA3A and GSK3β expression; re-myelination and axon sprouting	[84]
Rat sciatic nerve autograft model (PNS)	1 MHz	250 mW/cm^2^	20%	5 min/time, every other day	increased axonal regeneration rate; increased sciatic functional index; myelinated nerve fiber density upregulation	[74]
Mouse brain (CNS)	1 MHz	5 mW/cm^2^	20%	5 min per day for 3 consecutive days	Increase in DCX-positive cells; Upregulation of p-ERK expression	[87]
Mice Traumatic brain injury (TBI) (CNS)	1 MHz	500 mW/cm^2^	20%	15 min/day (administered as 3 sessions of 5 min each, with 5 min intervals), starting post-injury.	Reduced microglial activation, promoted M2 polarization; preserved hippocampal synaptic integrity; improved long-term neurological and cognitive function.	[93]
TBI (delayed treatment) (CNS)	1 MHz	500 mW/cm^2^	20%	Treatment initiated 3 or 6 h post-injury, administered daily for 15 min.	Improved body weight recovery, neurological severity scores, anxiety-like behavior, spatial working memory, and long-term learning and memory	[93]
Mice Traumatic brain injury (TBI) (CNS)	1 MHz	528 mW/cm^2^	20%	three 5 min sessions with 5 min rest intervals	Improved neurological function; Reduced brain edema, tissue loss, and neurodegeneration	[86]
Mice β-amyloid peptide (βA1-42)-induced Alzheimer‘s disease model (CNS)	1 MHz	800 mW/cm^2^	50%	6 min pulses per day; treatment began 24h after βA1-42 induction and continued for 17 days.	Reversed memory deficits; reduced levels of pro-inflammatory cytokines; increased anti-inflammatory cytokine	[92]

**Table 3:** Summary of piezoelectric materials for neural repair and their biological effect.

Material Composition	Stimulation Source	Target Cells	Main Biological Effects	Reference
PLLA/PEG electrospun APNF-NGC (aligned piezoelectric nanofibers)	LIPUS	Neurons (iPSC-derived motor neurons), Schwann cells	Directed axonal elongation; increased GAP-43; increased NGF/BDNF; enhanced Schwann cell migration; comparable to autograft in vivo	[108]
PVDF/PLGA electrospun outer layer (PVGA) + rGO/GelMA hydrogel inner layer with microgrooves	LIPUS	Schwann cells, PC12 cells	Directional cell migration via microgrooves; decreased ROS; decreased apoptosis; increased axonal regeneration (NF200); increased myelin thickness; increased CMAP/NCV; motor function recovery	[112]
SF/PEDOT conductive multi-channel cryogel + PVDF/PLCL piezoelectric outer film	Ultrasonic therapy	Schwann cells, PC12 cells	Increased SC proliferation & myelination (MBP, PMP22, NGF upregulated; NCAM downregulated); increased PC12 differentiation (GAP-43, NF-200 upregulated); increased angiogenesis (CD31); increased CMAP; increased SFI; increased myelin area	[107]
PHBV/PLLA/KNN nanowire composite film	Ultrasound	Schwann cells, macrophages, neurons	Increased CMAP amplitude; increased myelination; increased SFI; real-time nerve repair monitoring; biodegradable; wireless stimulation	[113]
BTNPs/P(VDF-TrFE) aligned electrospun nanofibers + pNIPAM hydrogel (NGF-loaded)	Ultrasound	PC12 cells, Schwann cells	Directed neurite extension; increased cell differentiation; US-triggered NGF release; increased S-100β, NF200; functional recovery in sciatic nerve defect	[110]
CNTs@GelMA/PLLA composite scaffold (aligned electrospun PLLA shell + conductive CNTs@GelMA hydrogel core)	Body movement/ultrasonic vibration	Schwann cells, DRG neurons	Increased Cell adhesion & elongation; increased neurite outgrowth; increased myelination; increased SFI; increased CMAP amplitude; increased muscle weight recovery	[114]
Aligned Fe_3_O_4_/PVDF electrospun nanofibers	External static magnetic field	RSC96 cells, DRG explants	Increased oriented cell migration; increased axon extension (up to 1720 μm); increased EGR2, CNTN2, Trpc2; increased calcium signaling; increased membrane potential variation; increased SFI; increased CMAP; increased muscle recovery	[115]
PCL/ZnO electrospun nanofiber (PZNF)	Mechanical deformation	Schwann cells, neurons	Increased NGF/VEGF expression; increased GRB2 expression; activation of RAS/MAPK pathway; rapid axon reconnection (<4 weeks); increased myelination	[116]
PVDF/PLCL/PEDOT composite film	Mechanical force or Ultrasound	Macrophages, Schwann cells	Increased M2 macrophage polarization; increased PI3K/AKT-Nrf2 pathway; increased HO-1; increased NGF/BDNF/SOX-10; downregulated NCAM; increased myelination and angiogenesis; immune modulation	[117]
PCL-β-glycine aligned composite nanofibers	Low-frequency mechanical vibration	Schwann cells (RSC96), PC12 cells	Increased myelination (S100); increased neurite outgrowth; increased cell alignment; increased motor function (99% recovery); increased CMAP amplitude (96% recovery)	[109]
PLA-HNA melt-spun fibers → twisted yarns → spiral-structured nerve conduits (SNCs)	Joint motion/mechanical deformation (in vivo)/US (in vitro)	Schwann cells (RSC96)	Increased cell proliferation, migration, alignment; increased S100B, NGF, NF-H, MBP, CD34; increased M2 polarization; increased PI3K-Akt/MAPK pathways; comparable to autograft in 10 mm defect	[118]
PVA/glycerol/BaTiO_3_ electrospun TP hydrogel outer layer + PCL/CNT electrospun PC fiber inner layer (bamboo-inspired bilayer)	Body motion (joint bending, walking)	Schwann cells, DRG neurons, HUVECs	Increased axon length (3.1-fold); increased myelin diameter (1.6-fold); increased angiogenesis (CD31, VEGF); increased NGF/GAP43; increased Ca^2+^ influx; SFI recovery	[111]

## Data Availability

No new data were created or analyzed in this study. Data sharing is not applicable to this article.
